# Case report: an atypical peripapillary uveal melanoma

**DOI:** 10.1186/1471-2415-14-13

**Published:** 2014-02-03

**Authors:** Li-Anne Lim, Cristina Miyamoto, Paula Blanco, Silvin Bakalian, Miguel N Burnier

**Affiliations:** 1 The Henry C. Witelson Ocular Pathology Laboratory, McGill University, 3775 University St., Room 216, Montreal, QC H3A 2B4, Canada

**Keywords:** Uveal melanoma, Monosomy 3, Fine needle biopsy

## Abstract

**Background:**

The treatment of uveal melanoma has seen a shift towards eye conserving treatments. Efforts have been made towards the identification of patients at high risk of metastatic disease with the use of prognostic fine needle biopsy, Monosomy 3 a risk factor for metastatic death thought to occur early in the development of uveal melanoma.

**Case presentation:**

We report a case in which an atypical optic nerve lesion was found to be a peripapillary primary uveal melanoma with distinct non-pigmented and pigmented halves on gross dissection and corresponding disomy 3 and monosomy 3 halves. The tumour demonstrated rapid growth with apparent transformation from disomy 3 to monosomy 3.

**Conclusions:**

These are clinical features that challenge the current concepts of the cytogenetic pathogenesis of uveal melanoma and demonstrate the potential problems and limitations of prognostic fine needle biopsy and molecular classifications.

## Background

The treatment of uveal melanoma has shifted towards eye conserving treatments, prognostic fine needle biopsy used to identify patients at risk of metastatic disease. Monosomy 3 is associated with a high risk of progression to metastatic death [1,2] and is thought to occur early in the development of uveal melanoma [2-4]. More recently, molecular classifications using gene expression profiling have been used, dividing tumours into those with a low metastatic potential (class I), those with a short term low metastatic potential, but higher mid to long term risk (class Ib) and those with short-term high metastatic potential (class II) [5].

We report a case in which an atypical optic nerve lesion, was found to be a peripapillary primary uveal melanoma with distinct non-pigmented and pigmented halves on gross dissection and corresponding disomy 3 and monosomy 3 halves. The tumour demonstrated rapid growth with apparent transformation from disomy 3 to monosomy 3, clinical features that challenge the current concepts of the cytogenetic pathogenesis of uveal melanoma and demonstrate the potential problems and limitations of prognostic fine needle biopsy and molecular classifications.

## Case presentation

**Figure 1:**
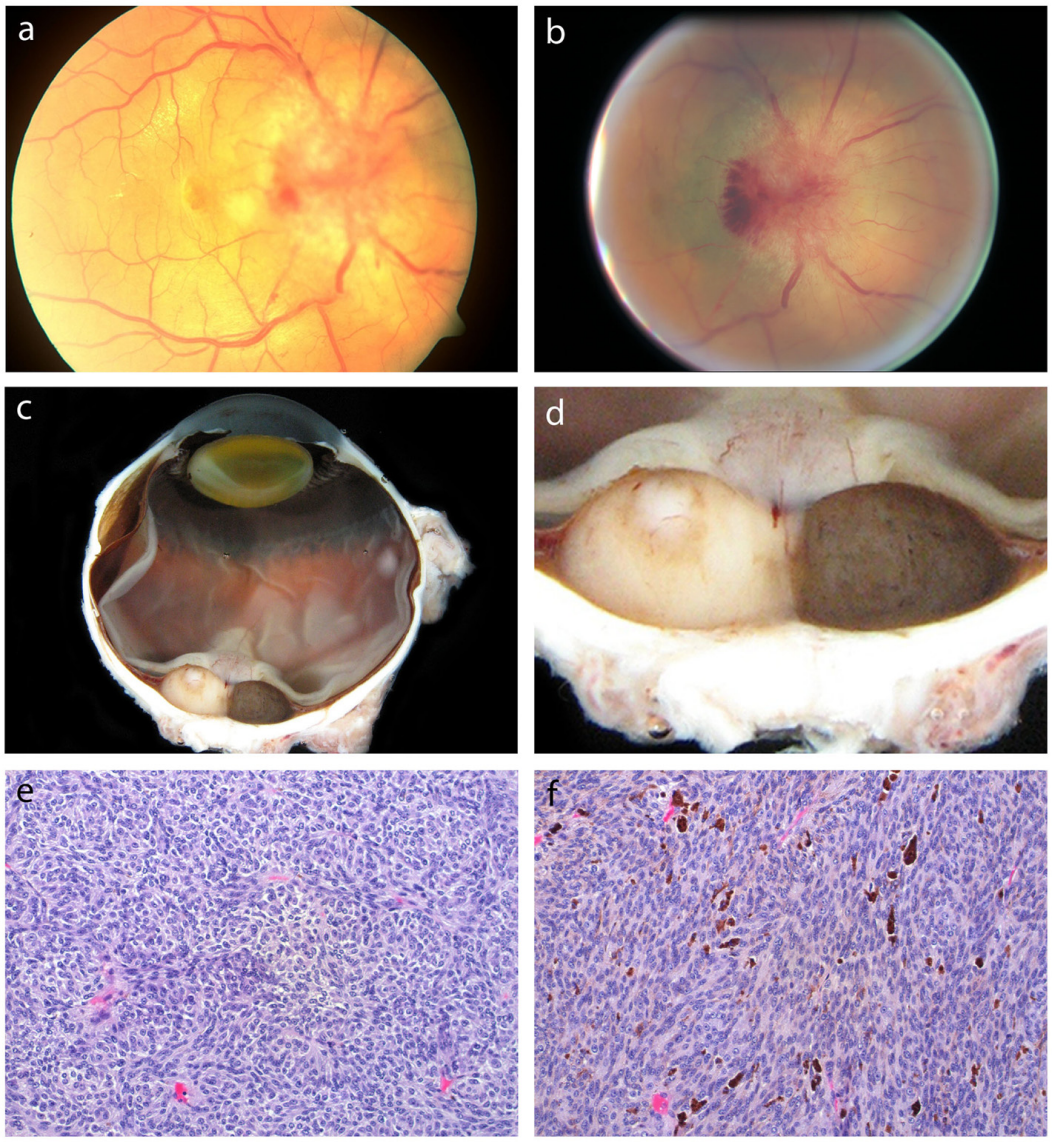
**Clinical and pathological findings. (a)** Initial dilated fundus examination: disc edema, hemorrhage and a serous retinal detachment. **(b)** Fundus examination 10 months later: increase in the size of the mass, with disc border hemorrhage and an adjacent ring of pigmentation. **(c) (d)** Gross pathology: uveal melanoma with distinct non-pigmented and pigmented halves. **(e)** Histopathological section of the non-pigmented half of the uveal melanoma, spindle cell type. **(f)** Histopathological section of the pigmented half of the uveal melanoma, of spindle cell type with melanin.

**Figure 2:**
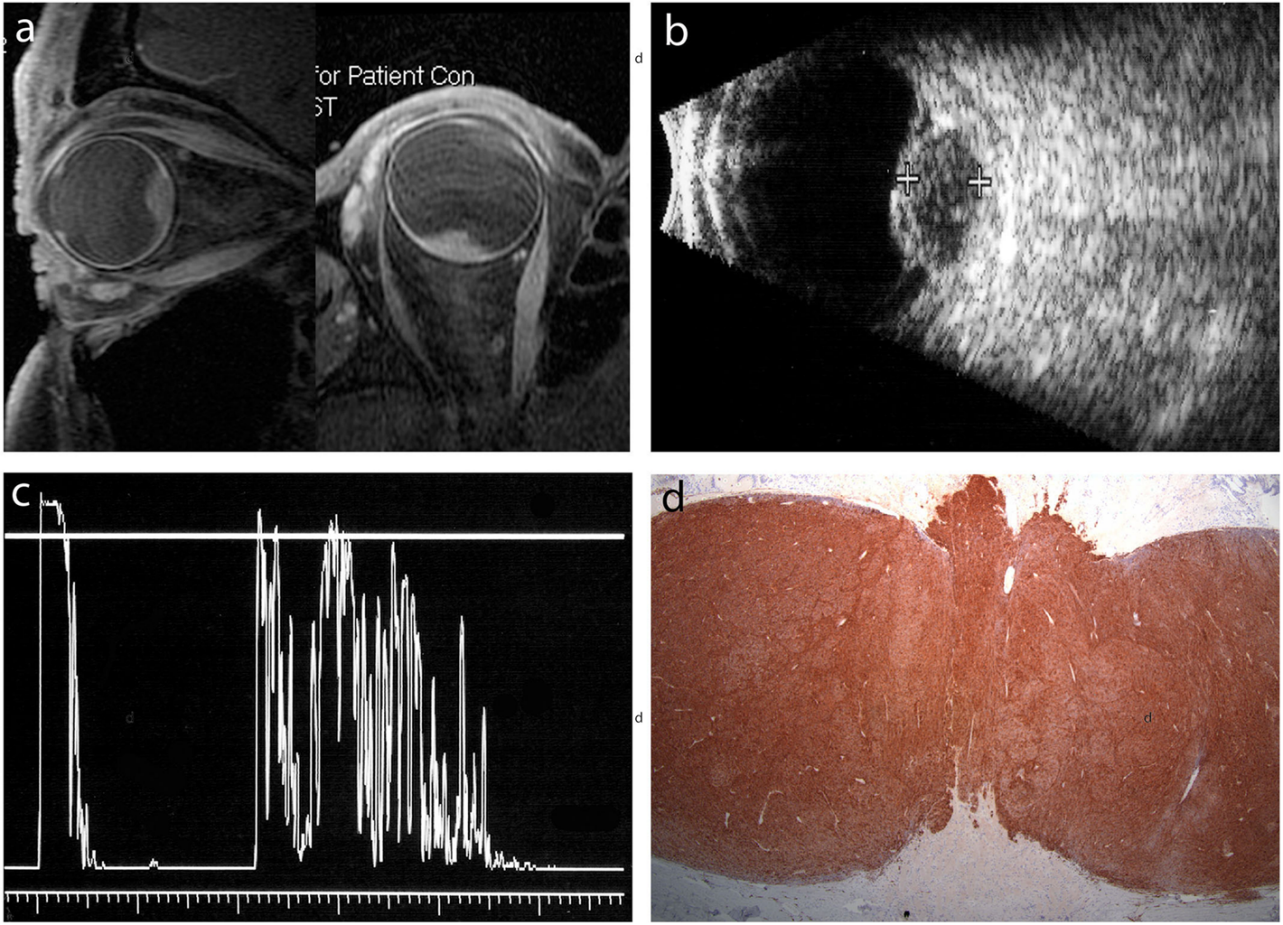
**Imaging and immunohistochemical findings. (a)** T1 weighted MRI of the globe: an elevated intraocular lesion at the posterior pole of moderately high signal. **(b)** B-scan ultrasound: dome shaped lesion at the posterior pole. **(c)** A-scan ultrasound: solid lesion at the posterior pole with low internal reflectivity and a kappa angle. **(d)** Immunohistochemistry: HMB-45 positive staining.

A 55- year- old man presented with a 4-year history of progressively worsening visual acuity in his right eye. His background medical history was otherwise unremarkable. On examination, visual acuity was 20/20 in the left eye and counting fingers at 1 m in the right eye. There was a right relative afferent pupillary defect, and a right central scotoma on fields to confrontation. Extraocular movements and intraocular pressure was within normal limits in both eyes and slit lamp examination was normal. On dilated fundoscopic examination, there was diffuse disc edema with marked disc hemorrhage and a related serous retinal detachment, findings suggestive of an optic nerve tumour (Figure 1a). Fluorescein angiography showed a well-vascularized mass at the optic nerve head, with fluorescein leak seen in late phase images. A provisional diagnosis of an optic nerve meningioma was given and the patient was referred for an MRI. MRI of the orbits however, showed an elevated intraocular lesion at the posterior pole with a moderately high signal on T1 weighted images suggestive of a peripapillary choroidal melanoma (Figure 2a). Diagnostic ultrasound with B-scan confirmed the presence of a dome shaped lesion 4.6 mm in thickness as well as a retinal detachment (Figure 2b). Standardized ultrasound with A-scan probe revealed a solid lesion with low internal reflectivity and positive kappa angle (Figure 2c). These findings confirmed the diagnosis of choroidal melanoma. At that time, the patient did not wish to proceed with further management and was subsequently lost to follow up. He did however represent 10 months later. The lesion appeared to have rapidly increased in size, with further disc hemorrhage at the temporal border from 7 to 11 o’clock and a ring of adjacent retinal pigmentation (Figure 1b).

Enucleation was performed. Gross pathology revealed a uveal melanoma arising in the area adjacent to the optic nerve with distinct non-pigmented and pigmented halves (Figure 1c, d). On histopathological examination, both regions were comprised of spindle cells (Figure 1e, f). The non-pigmented tumour however, showed no vascular loops or lymphocytes, while the pigmented tumor did. Both tumors were positive for HMB-45 and Melan-A immunohistochemistry (Figure 2d). Cytological examination of the non-pigmented tumour revealed no alterations on chromosomes 3 or 8, while the pigmented tumour contained monosomy 3 with alterations on chromosome 8 also.

## Materials and methods

The entire eye was formalin fixed, paraffin embedded (FFPE) and routinely processed and stained with hematoxylin-eosin (H&E). This tissue was obtained and managed in accordance with the guidelines of the declaration of Helsinki. Cytomorphology was assessed on H&E sections and categorized according to the modified Callender system [6]. Immunohistochemical stains Melan A and HMB-45 were used to confirm the diagnosis of uveal melanoma. Vascular loops were assessed using the PAS reagent without counterstaining. The presence of tumour infiltrating lymphocytes was assessed using immunohistochemical antibodies for B cells, L-26 (Dako) and T cells UCHL-1 (Dako). Significance was considered as more than 100 lymphocytes per 20 high-powered (43×) fields [7]. Fresh tumour tissue was taken for single nucleotide polymorphism analysis following enucleation, and further processed to assess for chromosomal abnormalities. DNA extraction from the samples was performed using commercially available isolation kits (DNeasy Kit; Qiagen, Germantown, MD) according to the manufacturer-suggested protocols. A labeling and detecting whole-genome genotyping sample kit (Illumina Human 660 W-Quad v1; Illumina San Diego, CA) was used to analyze for monosomy 3. Data analysis was performed using commercial data analysis software (GenomeStudio with KaryoStudio module; Illumina).

## Discussion

This case of uveal melanoma showed a lesion that underwent rapid growth, enlarging and developing a ring of pigmentation over a period of only 10 months. Furthermore, the seemingly aggressive portion of the tumour correlated with apparent transformation from a non-pigmented slow growing normal heterodisomy 3 tumour to a pigmented monosomy 3 tumor that also exhibited other poor prognostic factors including the presence of vascular loops and tumour associated lymphocytes. This is similar to one other case reported by Callejo et al. [8] in which an apparently quiescent melanoma suddenly enlarged, became necrotic, and apparently transformed from an indolent disomy 3 spindle cell uveal melanoma to an aggressive monosomy 3 epithelioid tumour. White et al. [9] also reported a similar case in which the uveal melanoma tumour showed grossly distinct pigmented and non-pigmented regions. These two regions however, were morphologically different, the pigmented area of the uveal melanoma composed of small epitheliod cells while the non pigmented area was comprised of large pleiomorphic epitheliod cells. This morphological heterogeneity corresponded with cytogenetic heterogeneity, the two areas showing different karyotypes, the pigmented tumor showing monosomy 3, and the non-pigmented tumour showing two normal chromosomes 3. DNA analysis of both pigmented and non-pigmented regions revealed loss of heterozygosity at all informative loci at chromosome 3, suggesting that duplication of the monosomy 3 chromosome had occurred (isodisomy 3) and that the non pigmented tumour had evolved from the pigmented tumour.

Isodisomy 3 is thought to occur in 5-10% of cases of uveal melanoma, and is prognostically equivalent to that of monosomy 3 status [10]. It is associated with the high risk class II expression signature and the development of metastasis, implying that chromosome 3 in monosomy 3 tumours, is defective at one or more tumour suppressor loci, this then being duplicated in isodisomy 3 tumours [10]. Intratumoural heterogeneity for chromosome 3 has also been previously described in tumour specimens [11-13], found to occur in 14-18% of uveal melanomas [14]. Evidence of subclone formation from analyses of harvested cells [2,9] and morphological heterogeneity corresponding with cytogenetic heterogeneity has also been reported by Sandinha et al. [11].

These cases suggest that monosomy 3 may arise at any stage during tumour development. As a result, this raises the question of whether small tumours and disomy 3 uveal melanomas should be treated early to prevent progression or evolution into a high grade lesion with more malignant potential. Furthermore, these cases in which there is clearly demonstrated intratumoral heterogeneity further emphasize its previously reported existence [14,15]. The limitations of prognostic fine needle biopsy are also highlighted; as aspirate material may not adequately represent the tumor in its entirety. Thus, the usefulness of this investigation as a prognostic test, especially in the scenario of a negative result for monosomy 3, may also need to be further considered.

More recently, the gene expression profiling assay has been shown to not be affected by intratumoral heterogeneity in a small sample of tumours [16], and in the recent Collaborative Ocular Oncology Group study [17], has been shown to be technically successful and to be the most accurate prognostic marker compared with clinical and histopathological factors, a class II signature more strongly associated with metastasis than any other prognostic factor including monosomy 3 [17]. However, it is important to note that although a statistically significantly small number of metastasis did occur in 3 patients (1.1%) with class I tumours compared with 44 patients (25.9%) with class II tumours at a median follow up of 17.4 months (p < 0.0001), metastases still do arise from a small number of class I tumours [17]. Therefore, errors relating to tissue sampling bias, tumour progression, and the unpredictable clinical behavior of uveal melanoma with delayed development of metastases as a result of tumour dormancy, support the need for longer term follow up data of the molecular classification to further validate its accuracy and reliability.

## Conclusion

We present an atypical peripapillary uveal melanoma that demonstrated rapid growth and apparent transformation into a monosomy 3 tumour, a case that challenges current concepts of the pathogenesis of uveal melanoma, and highlights intratumoural heterogeneity and sampling error as persistent sources of uncertainty. Although gene expression profiling appears to be unaffected by this, further long-term study is needed to assess the accuracy of this molecular classification.

## Competing interests

The authors declare that they have no competing interests.

## Authors’ contributions

MB and SB were clinicians involved with this case. SB and MB were involved with pathological processing and clinical assessment of the specimens. LL wrote the manuscript. LL, CM, PB, SB and MB analyzed the data and discussion. All authors read and approved the final manuscript.

## Pre-publication history

The pre-publication history for this paper can be accessed here:

http://www.biomedcentral.com/1471-2415/14/13/prepub
